# Video-feedback intervention for promoting social engagement in children with neurodevelopmental disabilities

**DOI:** 10.3389/fpsyg.2024.1504338

**Published:** 2025-01-08

**Authors:** Rosario Montirosso, Serena Micheletti, Camilla Pisoni, Eleonora Mascheroni, Elisa Scarano, Cecilia Naboni, Elisa Rosa, Annalisa Castagna, Margherita Bonino, Elisa Fazzi, Simona Orcesi

**Affiliations:** ^1^0-3 Center for the at-Risk Infant, Scientific Institute IRCCS Medea, Lecco, Italy; ^2^Unit of Child and Adolescence Neuropsychiatry, ASST Spedali Civili, Brescia, Italy; ^3^Neonatal Unit and Neonatal Intensive Care Unit, Fondazione IRCCS Policlinico San Matteo, Pavia, Italy; ^4^Department of Clinical and Experimental Sciences, University of Brescia, Brescia, Italy; ^5^Department of Brain and Behavioral Sciences, University of Pavia, Pavia, Italy; ^6^Neuropsychiatry and Neurorehabilitation Unit, Scientific Institute IRCCS Medea, Lecco, Italy; ^7^Department of Child Neurology and Psychiatry, IRCCS Mondino Foundation, Pavia, Italy

**Keywords:** neurodevelopmental disabilities, early intervention, video-feedback intervention, child engagement, emotional regulation, face-to-face still-face paradigm

## Abstract

**Introduction:**

Children with neurodevelopmental disabilities (NDs) display several developmental impairments across various domains that impact parent–child interactions, emphasizing the need for effective early interventions. This multi-centric study aimed to evaluate the impact of video-feedback intervention (VFI) on enhancing maternal behavior (i.e., sensitivity) and socio-emotional skills (i.e., engagement and emotionality) in children with NDs during normal or stressful interactions (i.e., the Face-to-Face Still-Face, [FFSF]) paradigm.

**Methods:**

A single-cohort design with pre-(T0) and post-(T1) intervention assessment was used to evaluate 37 mother–child dyads from three units in Northern Italy. The children, aged between 6 and 24 months, had a diagnosis of ND, including psychomotor delay (*n* = 26) and cerebral palsy (*n* = 5). The VFI was administered over 6 weeks, with each session focusing on improving parents’ developmental supporting behaviors and enhancing the quality of parent–child interactions. Using the Global Rating Scale coding system (GRS), child behaviors (engagement and emotionality) and maternal behavior (sensitivity) were assessed during the FFSF.

**Results:**

Analyses of variance (ANOVA) indicated significant improvement post-intervention in child engagement in the Reunion episode, with an increase in mother-directed gaze communicative gestures and positive vocalization. A paired sample *t*-test revealed that the mother’s *sensitivity* significantly improves between T0 and T1 during the Reunion phase. Moreover, a higher increase in sensitive maternal behavior during the Reunion phase was associated with greater child engagement at T1 during the Reunion episode. No significant changes post-intervention were observed in the emotionality of the child.

**Conclusion:**

The VFI effectively enhanced socio-emotional skills and maternal sensitivity during parent–child interactions, particularly in recovering from interactive disruptions.

## Introduction

### Background

Neurodevelopmental disabilities (NDs) are an umbrella term for conditions associated with impairment of the nervous system and include cerebral palsy, genetic syndromes, metabolic diseases, and severe brain injuries often associated with preterm birth (Ismail and Shapiro, 2019). According to the definition given by Morris et al. (2013), NDs are defined as a group of congenital or acquired conditions resulting from impairments in the brain and/or neuromuscular system, leading to functional limitations. These conditions can vary in presentation over time, occur independently or in combination, and encompass a wide range of severities and complexities. Their impact may involve challenges in movement, cognition, hearing and vision, communication, emotion, and behavior (Olusanya et al., 2018; Tassé et al., 2012).

Children with NDs typically face significant challenges in everyday socio-emotional competence, including a reduced understanding of social cues, difficulties with emotional regulation, and other related issues, which impact early parent–child interactions (Lipkin et al., 2020; Montirosso et al., 2020). These children tend to be less responsive and attentive, offering fewer vocal and emotional cues, exhibiting more irritability, and providing less clear social signals (Okimoto et al., 2000). Furthermore, children with NDs are particularly dependent on their caregivers for emotional support and for helping in dealing with social stress (Hauser-Cram and Woodman, 2016).

Parents of children with NDs frequently struggle to be responsive to their child’s needs, which includes effectively reading and responding to their signals, providing suitable stimulation, and keeping their attention engaged (Castagna et al., 2024). These parents may also adopt a more directive or intrusive approach, likely aiming to enhance opportunities for understanding and responding to their children’s unclear communicative signals (Guralnick et al., 2008; Venuti et al., 2009; Bornstein et al., 2012). In addition, they may experience high levels of emotional distress (Larkin et al., 2021), which can impact their ability to engage in sensitive and responsive interactions (Innocenti et al., 2013).

Recent studies emphasize that positive parenting—such as being accepting, warm, involved, sensitive, responsive, caring, and empathetic—has a beneficial impact on the functional outcomes of young children with NDs (Feldman and Baker, 2012; Spiker et al., 2002; Assel et al., 2003; Festante et al., 2019; Vilaseca et al., 2019b). More specifically, features of positive parenting include those that promote the connection between parent and child (e.g., maternal responsiveness and engagement during parent–child interactions), behavior regulation (e.g., turn-taking control), and respect for the child’s individuality (e.g., avoiding intrusiveness). In a meta-analysis including 14 studies and 576 participants, Dyches et al. (2012) found a significant relationship between positive parenting attributes and outcomes of young children with developmental disabilities (e.g., language and learning). Maternal sensitivity, scaffolding, and teaching behaviors have also been linked to improved developmental outcomes, including cognitive and language skills, in preschool-aged children with NDs (Vilaseca et al., 2019a). Finally, in providing interactive support, parents are key in offering cognitive stimulation during interactions, which has long-term benefits for children’s cognitive, language, and socio-emotional development, continuing into preschool and school years (Anderson et al., 2013; Innocenti et al., 2013; Totsika et al., 2020). Together, these findings underscore the critical importance of enhancing positive parenting to support children with NDs (Spittle and Treyvaud, 2016).

### Video-feedback as early intervention for children with NDs

Early parenting interventions are beneficial to improve the developmental outcomes of young children and limit some of the detrimental effects that the presence of NDs has on the quality of parent–child interaction (Morgan et al., 2021). The video-feedback intervention (VFI) is an early intervention designed to strengthen the parent–child relation by promoting sensitive and attuned parenting. Its primary aim is to indirectly enhance infants’ behavioral and socio-emotional competencies by improving parental sensitivity and understanding (Rusconi-Serpa et al., 2009). VFI helps parents observe themselves interacting with their child from an external perspective, enabling them to better understand and respond to their child’s unique communicative and interactive needs, which can be particularly challenging for those with NDs (Rusconi-Serpa et al., 2009). By reflecting on both positive interactive episodes and moments of interactive difficulties, parents are able to gain insights into their child’s behavior and shape their responses accordingly. This reflective process supports parents in enhancing their sensitivity, ultimately fostering more attuned and effective interactions with their children (Sealy and Glovinsky, 2016).

During mother–child interactions, children with NDs often encounter challenges in social engagement, characterized by reduced gaze orientation toward caregivers (Einfeld et al., 2001; Jahromi et al., 2008; Odding et al., 2006; Sigurdardottir et al., 2010) and unclear, ineffective, or nuanced social-communication signals (Festante et al., 2019). They also have difficulties displaying positive emotionality during mother–infant exchanges (Giusti et al., 2018). These characteristics can significantly affect maternal behaviors. Research shows that mothers of children with NDs often exhibit less sensitive caregiving behaviors (Azad et al., 2013), frequently missing subtle communicative signals from their child and responding less contingently. In contrast to mothers of typically developing children, these mothers are more likely to adopt directive approaches (Guralnick et al., 2008) or intrusive approaches (Blacher et al., 2013; Venuti et al., 2009). The VFI enhances parents’ ability to read and respond to their children’s signals, fostering greater sensitivity, effective regulation strategies, and the capacity to provide appropriate stimulation. By encouraging interactive exchanges, it promotes more attuned and supportive parent–child interactions, ultimately strengthening the child’s socio-emotional skills, including social engagement and positive emotionality (Bakermans-Kranenburg et al., 2003).

Recent evidence suggests that video-feedback intervention (VFI) specifically tailored for children with NDs has proven to be an effective approach that can enhance parent–child relationships and child’s developmental outcomes (Provenzi et al., 2020; Montirosso et al., 2025). Several VFI programs have been developed to respond to the specific challenges of parent–child relationships under diverse at-risk developmental conditions (Hoffenkamp et al., 2015; Høivik et al., 2015; Platje et al., 2018; Yagmur et al., 2014). For example, a VFI adaptation for use with parents of children with hearing impairments has been found to be effective in decreasing behavioral problems (James et al., 2013) and enhancing communicative skills (Glanemann et al., 2013). One study engaging 2- to 4-year-old children with developmental disabilities showed that VFI focused on parents’ empowerment was significantly associated with a reduction of aggressive and disruptive behaviors (Phaneuf and McIntyre, 2011). In addition, children with moderate-to-severe NDs, including Down syndrome, demonstrated improved vocal autonomy and interactive behaviors following VFI (James et al., 2013; Lam-Cassettari et al., 2015). A recent meta-analysis reported that programs involving joint observation of parent–child interactions by parents and practitioners, either in real-time or using video-feedback technologies, had a significant impact in reducing externalizing behaviors in children with NDs (Kei et al., 2024), including those with intellectual disabilities (Leung et al., 2022; Salisbury et al., 2022). In sum, the VFI has been found to be effective in enabling caregivers to respond contingently and effectively to their child’s behaviors, which in turn can promote socio-emotional skills, including the child’s engagement and regulatory capacities (Grumi et al., 2023).

### Child’s engagement in the context of socio-emotional interactive challenges

Caregiver–child interactions encompass both challenging moments and supportive phases that foster the child’s socio-emotional capacities. Research on face-to-face interactions has shown that early dyadic exchanges are not characterized by stable synchronization between partners but rather by phases of coordination followed by periods of mismatch and repair (Lavelli and Fogel, 2013). Accordingly, studies using the Face-to-Face Still-Face (FFSF) paradigm have documented that socio-emotional skills, which include both social engagement and regulatory capacities, become more evident when the child has to cope with challenging interactive exchanges (Fuertes et al., 2024).

During the FFSF, the child first experienced a 2-min period of normal face-to-face interaction (Play episode), followed by 2 min of maternal unresponsiveness (Still-Face episode), and concluded with a return to normal interaction (Reunion episode). Compared to the initial interactive episode, there is generally a reduction in positive emotionality and social engagement by communicative signals such as gazes toward the parent during the Still-Face episode (the so-called, *Still-Face effect*; Adamson and Frick, 2003; Montirosso et al., 2012). During the Reunion episode, children typically exhibit recovery from the distress elicited by the maternal Still-Face, characterized by an increase in social engagement (i.e., eye contact with mother) and positive emotionality (i.e., smiling), referred to as the *Reunion effect* (Adamson and Frick, 2003). Notably, several dimensions of parenting, such as affection and responsiveness, have been associated with the child’s ability to adapt to psychosocial challenge episodes of FFSF (Northrup et al., 2019). For example, maternal sensitivity to 9-month-old infants’ cues during the first episode of the FFSF was associated with infants’ more frequent positive bids to the mother during the caregiver unresponsiveness episode. Similarly, 4-month-old infants who exhibited more positive communicative behavior during the maternal emotional unavailability of the FFSF had mothers who were more sensitive and responsive to them during the Reunion episode. Moreover, children who exhibited lower negative emotionality in response to temporary maternal unresponsiveness had mothers who were attentive to their needs and adept at detecting their signals during normal face-to-face interactions (Grant et al., 2010). In addition, maternal contingent responding was associated with more positive affect in the child during the FFSF episodes (Lowe et al., 2012; Mcquaid et al., 2009). Thus, the use of the FFSF paradigm offers several advantages for comprehensively examining a child’s socio-emotional skills before, during, and after a social challenge caused by disruptions in interaction (Montirosso et al., 2015; Mesman et al., 2009).

## The current study

Although the FFSF has been previously used to assess children with NDs in a clinical setting (Giusti et al., 2018; Anceresi and Provenzi, 2023) and their parents (Miron et al., 2009; Provenzi et al., 2020), to the best of our knowledge, it has never been applied for assessing the impact of early parenting interventions aimed at promoting developmentally supportive parenting behavior in children with NDs. In the current study, we assessed the impact of VFI to enhance children’s socio-emotional skills from before (T0) to after (T1) the intervention. We used the video clips to offer caregivers a brief parenting intervention aimed at enhancing parents’ sensitivity to their child by helping them to observe and reflect upon the child’s behavior [for details see: Montirosso et al. (2020)]. Our first hypothesis was that VFI would lead to an improvement in the child’s socio-emotional skills during the FFSF paradigm in the post-intervention assessment. Specifically, we expected that intervention might also lead to children displaying more socio-emotional skills, operationalized by engagement and emotionality, both in Still-Face and in Reunion episodes. Previous evidence suggests that positive maternal behavior was associated with more social signals across FFSF (Mcquaid et al., 2009; Lowe et al., 2012). Accordingly, we expected that the child’s socio-emotional skill improvement would be related to higher levels of sensitive maternal behavior fostered by VFI.

## Methods

### Participants

From June 2020 to July 2023, 37 Italian mother–child dyads were recruited in three clinical units in Northern Italy: (1) Neuropsychiatry and Neurorehabilitation Unit, Scientific Institute IRCCS Medea, Bosisio Parini (*n* = 13), (2) Child Neurology and Psychiatry Unit, ASST Spedali Civili, University of Brescia, Brescia (*n* = 13), (3) Child Neurology and Psychiatry Unit, IRCCS Mondino Foundation, Pavia (*n* = 11). Children’s inclusion criteria were as follows: (i) diagnosis of ND (e.g., cerebral palsy and developmental delay); (ii) chronological age between 6 and 24 months (corrected age in case of preterm birth); (iii) absence of severe hearing (e.g., deafness) or visual (e.g., blindness) impairment. Mothers’ inclusion criteria were as follows: (i) age over 18 years old; (ii) good knowledge of the Italian language; (iii) not being a single parent; (iv) absence of a diagnosis of psychiatric disorder or intellectual disability. Parents were invited to participate in this research by assuring them that the participation would be entirely voluntary, and all parents provided written informed consent. The research project has been conducted in accordance with the Code of Ethics of the World Medical Association (Declaration of Helsinki, 7th revision, 2013), and the study was approved by the Ethics Committees of the Scientific Institute IRCCS Medea, Bosisio Parini, Italy (protocol 42/18), and of the participating hospitals.

### Procedure

The present study was part of a multi-center project (Montirosso et al., 2020), and the study protocol included three phases: pre-intervention assessment (T0), six sessions of VFI, and post-intervention assessment (T1). At T0, a 10-min unstructured mother–child interaction with a standard set of toys (i.e., ball, rattle, and building blocks) was recorded to set up the VFI sessions. Moreover, at T0 and T1, the FFSF procedure occurred to evaluate socio-emotional skills and maternal behavior. Mothers completed a socio-demographic survey (i.e., maternal socio-demographic characteristics). The video-recording interactions took place in ambulatory settings of the three units involved. The mothers and their children were welcomed into a quiet room. Upon arrival at the room observation, the mothers signed informed consent. After a brief phase in which the researcher explained the procedure to the mother and allowed the child to acclimate to the unfamiliar environment, the recording began.

### Face-to-face-still-face paradigm

The FFSF paradigm comprised three 2-min episodes: (1) a normal Play episode, during which mothers were instructed to play with her child as they usually do; (2) a Still-Face episode, during which the mother was instructed to maintain a still poker-face expression avoiding any interactive behavior toward the child; (3) a Reunion episode, during which mother and child resumed playing as during the Play episode. The FFSF was videotaped for later coding. Although the FFSF procedure has been mainly used with infants under 10 months of age (Mesman et al., 2009), previous studies have successfully employed the FFSF paradigm in typically developing children up to 30 months of age (Montirosso and Tronick, 2008; Weinberg et al., 2008). Moreover, the FFSF paradigm has been applied to the broader age range (6–36 months; Mesman et al., 2009; Provenzi et al., 2016), which included preschoolers with neurodevelopmental disorders (Ostfeld-Etzion et al., 2015).

### The video-feedback intervention

The VFI was focused on the unique needs of each dyad, and the intervention aimed to improve the mother’s ability to recognize and respond to her child’s signals, support attention and regulation, and promote the child’s social and cognitive development. The VFI was conducted over 6 weeks, with each session lasting 45 min. During these sessions, trained research psychologists conduct the VFI. In each session, the intervention began with the introduction of theoretical concepts and practical examples related to a specific topic (Table 1). Afterward, mothers were shown clips of the 10-min unstructured mother–child interactions recorded at T0, which highlighted behaviors connected to the topic at hand. Psychologists employed open-ended questions and reflective comments to encourage parental insights, guiding the discussion in a non-directive manner (Montirosso et al., 2025). A range of standard psychological interview techniques were used, such as validation, requesting examples, reformulating, verbalizing, reflecting, paraphrasing, using metaphors, and summarizing. Psychologists often use techniques such as information-giving, modeling, and positive reinforcement to encourage particular parenting behaviors (e.g., directing the child’s attention).

**Table 1:** Specific topics and content covered in the six VFI sessions.

Topic	Focus	Main aim	Examples of activities
Responsiveness	Reading/responding to signals	Improve maternal responsiveness to the child’s signals	Focus on reading the child’s signals, on turn-taking during the interaction, and on imitating and mirroring the infant’s expressions
Attention and engagement	Promoting dyadic engagement and triadic attention	Enhance the child’s attention toward the mother and shared objects	Focus on the interaction with the child, on the socio-communicative signals of both members of the dyads, and on episodes of dyadic engagement and triangulation
Regulation	Managing stress	Support the child’s stress management through self and maternal regulation	Recognize the infant’s stress signals and any attempts at self-regulation, as well as the strategies the parents can implement to promote her/his emotional regulation and stability
Stimulation	Adjusting sensory input	Identify and modulate appropriate sensory stimulation for the child	Focus on the different channels of stimulation (e.g., tactile, visual, and auditory) and on the modulation of the intensity and frequency of stimulation. Understand which channels are preferred by the child
Encouragement-teaching	Supporting exploration and autonomy	Foster the child’s cognitive development, attention, and exploratory behaviors	Capture and harness the child’s attention, support exploration, follow the child’s interest, make activities accessible, and provide encouragement
Parental perspective	Emotional tone, mental representations, physical contact	Explore the mother’s emotional state, beliefs about the child, and physical interaction	Recognize own thoughts, emotion, expectation, and focus on the mental representations of one’s own child and oneself as parent. Try to take the child’s perspective

### Measures

#### Socio-demographic variables and child data

Socio-demographic variables were collected, and *ad hoc* questionnaires were filled out by mothers. The collected information included the age and gender of the child, maternal age, and maternal educational level. Child data, including diagnosis and equivalent age, were obtained from medical records.

##### Coding of child’s and mother’s behavior

The Global Rating Scale (GRS; Fiori-Cowley et al., 2000) coding system was used to assess a child’s socio-emotional behavior during Play, Still Face, and Reunion episodes and the mother’s behavior during Play and Reunion episodes. The GRS is a useful tool for examining the child and the mother’s behavior and their interactions (Murray et al., 1996). It has shown good reliability and validity across diverse samples (Murray et al., 1996), and has already been used with the FFSF paradigm (Williams and Turner, 2020). The original version of the GRS evaluates three behavioral dimensions of the child (*engagement*, *activity level*, and *emotionality*) and four behavioral dimensions of the mother (*sensitivity*, *intrusiveness*, *remoteness*, and *level of depression*). In the present study, to address the research aims and align with the characteristics of the FFSF paradigm, two child behavioral dimensions (*engagement* and *emotionality*) and one maternal behavioral dimension (*sensitivity*) were assessed. Each dimension was rated on a 5-point scale, with 5 indicating *optimal* behavior and 1 indicating *poor* behavior.

More specifically, the child’s behavior coding included the following:

- *Engagement* measures the child’s involvement in moments of mother-directed gaze and communicative exchanges, such as pre-speech gestures, and limb movements in response to the mother’s actions, vocalizations, and smiles. It also considered vocal exchanges, considering high-pitched, happy, and communicative vocalizations directed toward the mother. A high score is a sign that the child is more actively engaged with the mother and was given to children who frequently looked at their mother and were communicative and vocal; a low score indicates less level of engagement and was given to those who showed little or no mother-directed gaze and who were non-communicative.
- The child’s *emotionality* measures the level of pleasure, smiling, and laughter exhibited by the child during interactions with the mother. A high score indicates a higher level of positive emotionality and was given to children who were actively happy for much of the interaction, frequently positively vocalizing and smiling often; a low score indicates less positive emotionality and greater distress and was given to children who rarely smiled or vocalized and showed marked signs of distress.

Mother’s behavior coding included the following:

- The mother’s *sensitivity* measures the degree to which the mother attunes to her infant’s cues, responding in a manner that is appropriately aligned with the infant’s behavior while displaying warmth and acceptance toward the child.

Two independent coders for each unit coded the child’s *engagement*, the child’s *emotionality*, and the mother’s *sensitivity*. To reach a high level of reliability, coders underwent specific training to reach an interrater agreement equal to or greater than 80%. The training was conducted, supervised, and verified by an expert coder who already had reliability concerning the coding system. Any uncertainties in rating were resolved through discussion with expert coders. The child’s behavioral coding was checked for possible differences among Units, and no significant differences emerged (0.075 < *p* < 0.194). The recorded mother–child interactions were coded by research assistants who were blinded to the study aims and the study session (i.e., T0 or T1).

### Statistical analysis

#### Child socio-emotional behavior during the FFSF

One-way ANOVAs for continuous variables and the χ^2^ tests for categorical variables were used to compare socio-demographic variables in the three different Units. The preliminary check was conducted to test the possible relation between a child’s equivalent age and a child’s behavior. To determine whether child behavioral response varied in the different episodes of the FFSF paradigm (Play, Still-Face, and Reunion) and in relation to pre- and post-intervention (T0 and T1), two repeated-measure ANOVAs were performed with the child’s *engagement* and child’s *emotionality* as dependent variables. *Post-hoc* univariate comparisons were then run using a paired sample *t*-test.

#### Association between child socio-emotional behavior during the FFSF and maternal sensitivity

Preliminary analyses, including repeated-measures ANOVA and paired sample *t*-tests, were conducted to examine potential differences in maternal *sensitivity* between the Play and Reunion episodes at T0 and T1. Subsequently, two delta variables were calculated to quantify the changes in maternal *sensitivity* from T0 to T1 during the Play and Reunion episodes, named *Δ-sensitivity-Play* and Δ-*sensitivity-Reunion,* respectively. Finally, to examine potential associations, a series of correlations (Pearson’s correlations) were run between the child’s *engagement* and the child’s *emotionality* and maternal *sensitivity*. In particular, it was tested whether changes in maternal sensitivity (Δ*-sensitivity*) were associated with changes in child socio-emotional skills from T0 to T1 (i.e., Δ-*engagement*). Importantly, these analyses focused exclusively on the child behaviors that demonstrated significant improvement from T0 to T1.

## Results

### Descriptive statistics

Of the 37 mother–child dyads, 33 mothers followed the entire VFI and 31 mother–child dyads completed the FFSF at T0 and T1. Analysis showed no significant differences in socio-demographic characteristics between the children who were included in the final sample and those who were excluded due to incomplete participation or missed assessments. Nine dyads (29.0%) underwent VFI at IRCSS Eugenio Medea, 12 dyads (38.7%) at the University of Brescia, and 10 dyads (32.3%) at IRCCS Mondino Foundation. No significant differences in children and maternal characteristics emerged among Units. Socio-demographic variables for the final sample and statistical comparison among Units are reported in Table 2.

**Table 2:** Children and maternal characteristics and statistical comparison among Units

	***Descriptive*** ***statistics***			***Test***
***Characteristics***	***N***	**%**		
Child’s gender				
Female	16	48.4		*χ^2^* = 3.02. *p* = 0.221
Male	15	51.6		
Main diagnosis				
Psychomotor delay	26	83.9		*χ^2^* = 3.60. *p* = 0.464
Cerebral palsy	5	16.1		
	***Mean***	***SD***	***Range***	***Test***
Child’s chronological age (months)	16.58	5.77	6-25	*F* = 2.15 *p* = 0.135
Child’s equivalent age (months)	10.68	3.74	4-17	*F* = .85 *p* = 0.437
Mother’s age (years)	34.19	5.60	24-49	*F* = 1.41 *p* = 0.265
Mother’s education (years)	13.33	3.08	8-18	*F* = 1.38 *p* = 0.268

### Child socio-emotional behavior during the FFSF

**Figure 1:**
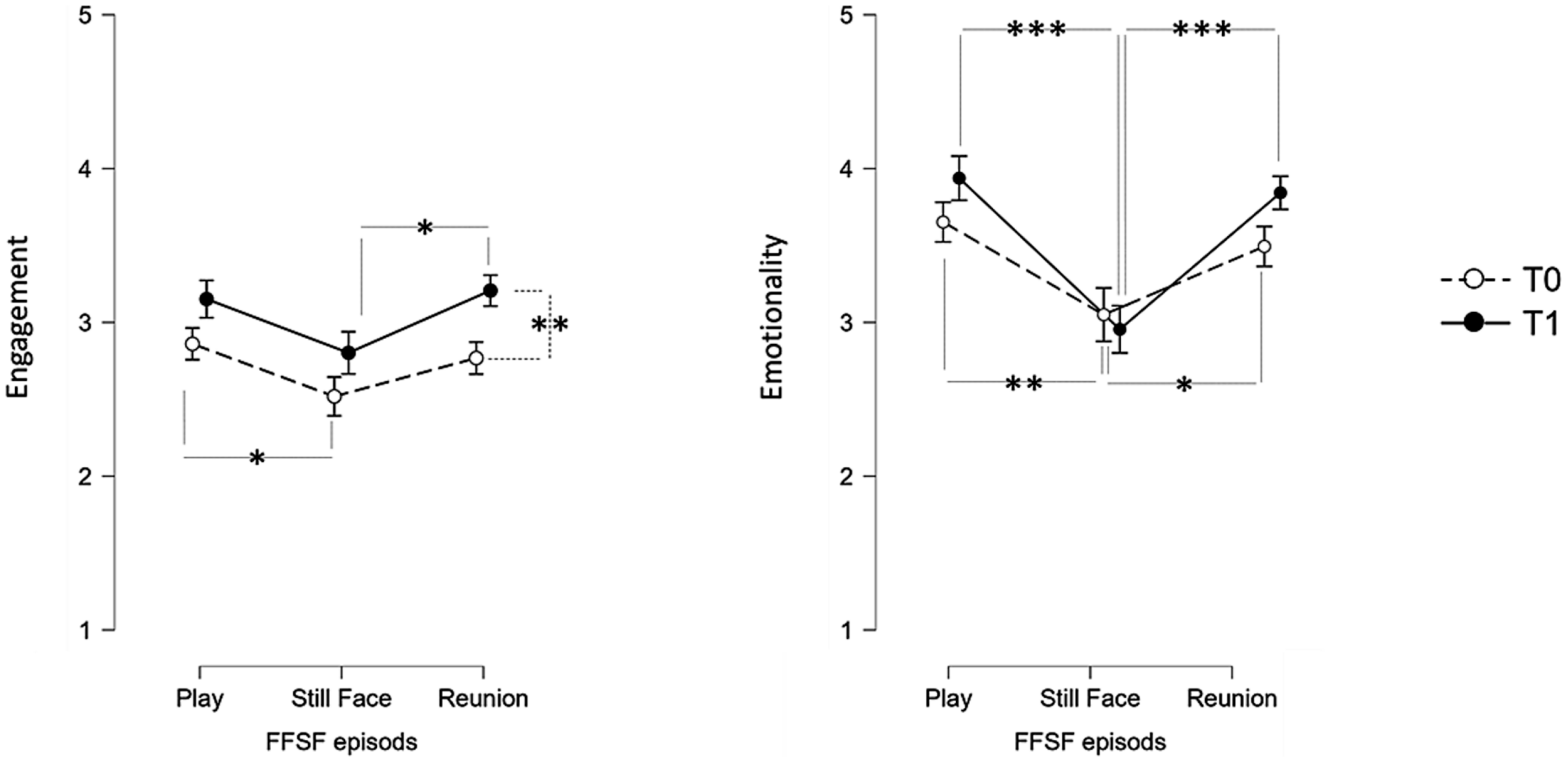
Differences between T0 and T1 in child behavioral response during the FFSF. *p < 0.05; **p < 0.01; and ***p < 0.001.

*Engagement*. Across the FFSF episodes, significant change emerged (*F* = 8.83, *p* < 0.001, *η*^2^ = 0.227) (see Figure 1 for means and standard errors). At T0, paired sample *t*-test revealed that the child’s *engagement* significantly decreases from Play to Still-Face [*t* = 2.19, *p* = 0.037, Cohen’s *D* = 0.393, IC 95% = (0.024 ÷ 0.775)], indicating the typical *Still-Face effect*; no significant differences were found between Still-Face and Reunion [*t* = −1.88, *p* = 0.070, IC 95% = (0.023 ÷0.736)], suggesting a *Reunion effect* (Mesman et al., 2009). At T1, no differences were found between Play to Still-Face [*t* = 2.01, *p* = 0.053, Cohen’s *D* = −0.337, IC 95% = (−0.697 ÷ 0.028)], but a significant increase between Still-Face and Reunion emerged [*t* = −2.35, *p* = 0.025, Cohen’s *D* = −0.422, IC 95% = (−0.787 ÷ − 0.051)]; these results suggest that after VFI children exhibited a less pronounced *Still-Face effect* and no *Reunion effect*. Furthermore, ANOVA revealed a main effect between pre- and post-intervention (*F* = 15.23, *p* < 0.001, *η*^2^ = 0.337). Univariate analyses yielded a significant increase in child’s *engagement* between T0 and T1 in *Reunion* (*F* = 9.17, *p* = 0.005, *η*^2^ = 0.234), confirming a more positive children’s social involvement during the recovery episode after the VFI. Moreover, differences in the *Still-Face effect* between T0 and T1 were highlighted by the significant interaction effect that emerged between FFSF episodes and pre−/post-intervention (*F* = 5.29, *p* = 0.008, *η*^2^ = 0.150).

*Emotionality*. ANOVA indicated that across the FFSF episodes, significant change emerged (*F* = 16.92, *p* < 0.001, *η*^2^ = 0.530) (see Figure 1 for means and standard errors). Both at T0 and T1, paired sample *t*-test revealed that child *emotionality* decreases from Play to Still-Face [T0: *t* = 2.80, *p* = 0.009, Cohen’s *D* = 0.503, IC 95% = (0.125 ÷ 0.873); T1: *t* = 4.66, *p* < 0.001, Cohen’s *D* = −0.840, IC 95% = (0.424 ÷ 1.25)] and increases from Still-Face to Reunion [T0: *t* = −2.34, *p* = 0.026, Cohen’s *D* = −0.421, IC 95% = (−0.789 ÷ −0.050); T1: *t* = − 5.48, *p* < 0.001, Cohen’s D = −0.984, IC 95% = (−1.41÷ −0.548)]. Overall, the findings suggested a *Still-Face effect* and a *Reunion effect* across FFSF both in T0 and T1. No other main or interaction effects emerged, indicating that no differences in the child’s *emotionality* were found in the pre−/post-intervention comparison.

### Association between child socio-emotional behavior during the FFSF and maternal sensitivity

ANOVA indicated no significant change across the FFSF episodes for the mother’s *sensitivity* (*F* = 3.04, *p* = 0.075, *η*^2^ = 0.102). Paired sample *t*-test revealed that mother’s *sensitivity* significantly improved between T0 and T1 during the Reunion episode [*t* = −2.15, *p* = 0.040, Cohen’s *D* = −0.377, IC 95% = (−0.730 ÷ −0.018)] but not in the Play episode [*t* = −1.05, *p* = 0.304, Cohen’s *D* = −0.188, IC 95% = (−0.542 ÷0.169)]. A significant correlation emerged between *Δ-sensitivity-Reunion* and child *engagement* at T1 [*r* = 0.440, *p* = 0.019, IC 95% = (0.080 ÷ 0.680)]. This result indicates that a higher increase in sensitive maternal behavior during the Reunion episode was associated with greater child *engagement* at T1 during the Reunion. We have also controlled whether changes in maternal *sensitivity* from T0 to T1 during Reunion episodes (i.e., Δ*-sensitivity-Reunion*) were associated with changes in children *engagement* from T0 to T1 (i.e., Δ*-engagement-Reunion*). A significant correlation emerged [*r* = 0.384, *p* = 0.043, IC 95% = (0.013 ÷ 0.662)].

## Discussion

In the current study, we assessed the impact of VFI on enhancing socio-emotional skills in children with NDs during normal and challenging interactive exchanges using a pre-post intervention design. We found that in the post-intervention assessment, children exhibited a less pronounced *Still-Face effect* and an enhanced recovery at the resumption of normal interaction after the maternal unresponsiveness. Specifically, both in the Still-Face and Reunion episodes, children exhibited increased mother-directed gaze, communicative gestures, and positive mother-directed vocalizations. According to previous studies, our findings suggest that VFI has fostered positive and sensitive parenting, which in turn promoted children’s socio-emotional behavior (Northrup et al., 2019; Reck et al., 2023). This interpretation is supported by evidence that compared to the pre-intervention phase, maternal sensitivity was higher during the Reunion episode in the post-intervention phase. Moreover, consistent with previous research (Gunning et al., 2013), maternal sensitivity was found to be associated with more child engagement during the recovery episode of the FFSF. Specifically, our findings are in line with those of Conradt and Ablow (2010), who reported that in the Reunion maternal and child behavior were related, suggesting that maternal sensitivity during distress provides a crucial contribution to support child socio-emotional skills. More broadly, our observations of concurrent associations between maternal sensitivity and child engagement support social development models that highlight the significance of positive parenting behaviors as a key factor in the dyadic exchanges, even in the presence of NDs (Giusti et al., 2018). We speculate that the VFI improved parenting qualities, such as parents’ capacities to read and respond to their child’s signals, fostering more attuned exchanges. This interpretation is in line with the Mutual Regulation Model (MRM; Gianino and Tronick, 1988). The MRM proposes that parent-infant interactions are shaped by a reciprocal exchange of communicative signals, which both the infant and caregiver use to coordinate their interaction and handle the social challenge caused by typical mismatching moments (Tronick and Beeghly, 2011). Accordingly, our results seem to suggest that interactive pattern which combines highly sensitive mother paired with a child who exhibits more communicative bids might promote the child’s ability to recover from challenging interactive exchanges (i.e., still-face episode). In sum, the findings underscore the potential of parenting early intervention to enhance the dynamic communicative processes central to effective caregiver-child interactions in children with NDs (Seifer et al., 1991) and, positively influence the infant’s ability to recover from a relational stress.

As for emotionality, consistent with previous research (Barbosa et al., 2018, 2019), the FFSF procedure elicited declines in the affective state from the initial Play to the Still-face episode, followed by elevation during the recovery episode of the FFSF at both assessment time points. Unexpectedly, we failed to find differences in emotionality between pre- and post-intervention. Although previous studies indicate that maternal sensitivity appeared to be substantially associated with emotional regulation (Mesman et al., 2009), we did not find a less pronounced *Reunion effect* in children with NDs after the intervention. This seems to suggest that children seem to profit less from VFI in terms of emotional regulation. One reason for the lack of evidence may be that emotional regulation in children with NDs is multifaceted (Hauser-Cram and Woodman, 2016). Consequently, it might require more targeted interventions to address the complexities of emotional distress and recovery. Moreover, changes in maternal behavior may require more time to affect a child’s emotional regulation than to impact child engagement. Therefore, the short time frame of follow-up assessment might be not enough to capture changes in a child’s ability to modulate emotional regulation, which would require a more gradual acquisition. Further research is needed to explore how VFI or other early interventions can more effectively impact emotional regulation in children with NDs. It may be beneficial to investigate additional strategies or combined approaches that specifically target emotional regulation, alongside improvements in engagement and interaction quality, to better support the developmental needs of children with NDs.

This study has several limitations that should be acknowledged. First, the use of a single-cohort design limits our ability to assess the efficacy of the VFI without a comparison group undergoing a different intervention. Including a control group in future studies would provide more robust insights into the effectiveness of VFI. Nevertheless, previous research evaluating the impact of VFI on parental skills and child outcomes in at-risk populations—similarly lacking a control group as in our study, has also demonstrated positive results (Phaneuf and McIntyre, 2007; Ousley et al., 2022). In addition, while the sample size in this study is comparable to that of previous research (Benzies et al., 2013; Kim and Mahoney, 2005), it remains relatively small. Importantly, the study sample comprised children with diverse ND diagnoses, resulting in substantial clinical variability that could impact both infants’ and parent’s behaviors, which may lead to differing effects of the VFI. Nonetheless, this heterogeneity reflects the diverse population commonly encountered in neuropsychiatry clinics addressing ND, aligning with the study’s objective to evaluate VFI within this broader clinical context. The relatively small sample size limited our ability to perform subgroup analyses. Future research should consider exploring differences across specific ND conditions and severity levels to better understand the intervention’s impact. Third, this study focused on changes in children’s interactive behaviors during the FFSF paradigm immediately following the intervention. Future studies should include follow-up assessments to evaluate the medium- and long-term effects of VFI on maternal sensitivity and infants’ behavior, particularly focusing on child emotional regulation. Future research should also examine the lasting impacts of VFI on child ongoing socio-emotional developmental trajectories. Fourth, a further limitation of this study is the lack of evaluation of mothers’ mental wellbeing and stress levels, which are critical factors influencing the quality of interactions with children, particularly those with NDs. The lack of these assessments limits the understanding of how the VFI might influence maternal wellbeing and, consequently, parenting behavior and practices. Future research should include these variables to provide a more comprehensive understanding of mother–child interactions.

Despite these limitations, our study shows the applicability of VFI in mothers of children with NDs, providing further evidence for the importance of implementing targeted parenting support in this population. In particular, this study highlights the relevance of VFI in the design of early interventions to support the developmental outcomes of children with NDs during their early years. Promoting the development of infants and young children through the active involvement of parents should be a priority for children with NDs (Guralnick, 2005; Schuster and Fuentes-Afflick, 2017). Therefore, we advocate for the increased promotion of early interventions targeting the parent–child system within healthcare services from the very first stages of a child’s life to maximize their effectiveness and ensure benefits for both families and healthcare systems (Doyle et al., 2009). In other words, given the impact of parental behavior on children’s socio-emotional skills, early parenting support warrants particular attention not only from healthcare practitioners but also from policymakers within the context of early rehabilitative interventions.

Overall, our findings indicate that maternal VFI is associated with improvements in children’s interactive behaviors, even during moderately stressful social interactions. While the results suggest that parental VFI is more effective in enhancing communicative abilities than emotional regulation in children with NDs, this intervention has the potential to strengthen the mother–child dyad’s ability to deal with interactive stress, which is a critical factor for positive developmental outcomes (Leclère et al., 2014). Our findings suggest potential long-term benefits of VFI for children with NDs. Research has shown that early positive mother–child dyadic relationships are prospectively associated with improved social competence over time (Fenning and Baker, 2012). By enhancing the quality of these interactions, VFI has the potential to support lasting improvements in children’s socio-emotional skills, facilitating integration into social and educational settings during preschool and school years. For these reasons, VFI protocols targeting parents of children with NDs should be integrated into clinical practice and offered to families alongside other validated individualized early interventions directed at children. These combined approaches should aim to promote both the social development of children and the emotional wellbeing of parents.

## Data Availability

The raw data supporting the conclusions of this article will be made available by the authors, without undue reservation.
